# Promoting Low-Carbon Purchase from Social Norms Perspective

**DOI:** 10.3390/bs13100854

**Published:** 2023-10-18

**Authors:** Dapeng Liang, Yang Fu, Mengting Liu, Jiayin Sun, Hongyan Wang

**Affiliations:** 1 School of Economics and Management, Harbin Institute of Technology, Harbin 150001, China; 2 School of Humanities, Social Science and Law, Harbin Institute of Technology, Harbin 150001, China; 3 School of Mathematical Science, Heilongjiang University, Harbin 150080, China

**Keywords:** descriptive norms, injunctive norms, low-carbon purchase (individual behavior, policy implementation, behavior experiment)

## Abstract

The importance of individual consumption behavior in a low-carbon economy is gradually recognized. Social norms have a significant effect on individual purchase behavior. However, the influence mechanism of social norms still needs more research. We conducted two behavioral experiments to explore the specific factors: first, the effect of descriptive norms on personal low-carbon consumption behavior through feedback information, and second, a comparison with injunctive norms, focusing on the impact of the normative focus shift brought by punishment represented by the policy implementation. The results show that social norms can effectively promote individual low-carbon consumption through feedback and high policy implementation efficiency. In particular, after effective policy implementation becomes an inherent element of injunctive norms, injunctive norms are activated and become the focus of norms, significantly improving the purchase rate of low-carbon goods.

## 1. Introduction

Due to the relationship between individual purchasing behavior and a low-carbon economy, personal low-carbon consumption behavior should be scrutinized. Inappropriate spending habits and consumption patterns of inhabitants are primarily to blame for environmental deterioration [1]. After people noticed that environmental deterioration had an impact on their lives, they began to use green products to protect the environment [2]. Consumers’ awareness of green consumption and willingness have increased [3]. Statistics show that green consumption, including low-carbon consumption, is increasing in both developed and developing countries [4]. Public involvement is crucial for effective environmental preservation [5]. Low-carbon consumption by individuals has gradually become an essential part of the low-carbon economy development. Therefore, individual carbon emissions have a critical impact on emission reduction. The 2015 Paris Agreement discussed the importance and emergence of emission reduction in recent years, which has come to the foreground [6]. The Chinese government has proposed a series of policies to develop a low-carbon economy [7]. The carbon emission reduction target was presented in the 14th Five-Year Plan for national economic and social development. The government has also raised the climate action goals for 2020 to 2030 [8]. If we want to achieve emission reduction goals better and faster, further research on individual low-carbon consumption behavior is an effective way.

In the study of behavior, as rules or group standards of behaviors and attitudes [9], social norms play an essential role in explaining and comparing behavior [10]. Social norms have a significant effect on prosocial behavior, including the action of descriptive norms in saving energy, water conservation, and recycling [11,12,13] and the performance of injunctive norms, such as carbon tax policy [14]. Farrow et al. (2017) reviewed nearly 100 studies on the intervention of social norms in pro-environmental behavior, including energy use, recycling, green consumption, etc. [15]. In their inventory of relevant experimental economics and social psychology literature, the social norm intervention in research of the first kind focuses on individuals’ perceptions of descriptive and injunctive norms, and in studies of the second kind, individuals are given factual information about norms [15]. Social norms can change behaviors effectively, but the mechanisms therein remain researched [16]. Opp (1979) noticed the importance of social norms in both the information-norm relationship and the norm-behavior relationship [17]. However, some important information about the social norm intervention has not been discussed in detail, such as information pertinent to policy efficiency or feedback. Individuals may perceive the stimuli of others’ behavioral feedback information without awareness. The levels of policy efficiency may have an effect on the injunctive norm. Studying these relevant factors from a micro-scale perspective is necessary to provide theoretical and empirical support for developing a low-carbon economy in China. Moreover, previous research about social norms is largely based upon survey studies and field experimental studies that investigate in descriptive and injunctive dimensions [15]. As feedback information and policy efficiency are difficult to measure with previous research methods, we used behavioral experiments to collect data.

In this paper, we provide full and accurate evidence on the effect of social norms on personal low-carbon purchase behavior. We conducted behavioral experiments to present the changes in individual purchase decisions when they noticed the social norms. By simulating the situation when shopping for goods, we allowed subjects to perceive different information about descriptive and injunctive norms in the experimental situation and analyzed the changes in norms on their decisions through their final purchase decisions.

This paper proceeds as follows: Section 2 provides background information and some definitions, and then outlines the hypotheses of the experiments. Section 3 presents the experiment itself. Section 4 presents the results and a Bayesian multinomial logistic regression used for analyzing the data. Section 5 discusses the implications of the results. Section 6 makes some conclusions.

## 2. Literature Review

### 2.1. Social Norms and Low-Carbon Purchase

Low-carbon consumption behavior is defined as a specific type of consumer behavior in which individuals consciously engage in daily activities aimed at reducing greenhouse gas emissions in response to the global climate crisis [18]. It encompasses the entire process of daily consumption, including purchasing (e.g., buying energy-efficient products) [19], usage (e.g., turning off appliances when not in use) [20], and recycling (e.g., recycling reusable items) [21]. Namely, it comprises three dimensions: low-carbon purchasing behavior, low-carbon usage behavior, and recycling behavior.

The impact of macro-level factors such as energy systems, consumption policies, and government promotion of innovative energy technologies on residents’ low-carbon consumption behavior is objectively present. However, when individuals are in complex and dynamic environments, it is often micro-level factors that play a decisive role in shaping their intentions and behaviors towards low-carbon consumption. Existing research largely focuses on achieving energy efficiency goals through improving energy use efficiency, while paying insufficient attention to the individual behavioral aspects of residents.

Recent studies indicate that social norms play a significant role in behavior [22]. Social norms, which have social rewards or punishments for performing security, occur in agreement with the behavior standards of community members [23]. Social norms influence individual pro-environmental behaviors [24,25] and have a positive impact, especially with regard to global environmental issues [26]. Many studies of consumer norms on individual green consumption have been proposed [27]. In environmental protection, social norms are beliefs that motivate individuals to be consistent with others and a code of conduct that can be accepted or not by group members [28]. They are widely used to study prosocial behavior, such as promoting green purchasing [6,29,30], energy conservation [31,32,33], and recycling [34,35]. In previous research, the factors influencing residents’ low-carbon consumption behavior were divided into three main categories: individual factors, family factors, and situational factors [36]. Social norms belong to the category of situational factors. One of the most predominant theories about social norms, the focus theory of normative conduct, considers that social norms can be divided into descriptive and injunctive norms [37]. The descriptive norms refer to typical or ordinary things that most people do. The concept of injunctive norms focuses on what ought to be done, like rules or beliefs. The focus theory of normative conduct holds that people show good behaviors, such as protecting the environment, because social norms affect their consciousness, attitude, and purpose [38]. Present studies indicate that descriptive norms have an influence on individuals through the behaviors of most people. Injunctive norms lead people to do good things by emphasizing what they should do [37,39]. In addition, descriptive norms and injunctive norms can exist with different guiding effects simultaneously. People will be inclined toward the norms that they focus more attention on when the effects of these two norms are conflicting [37,40].

#### 2.1.1. Descriptive Norms

Compared with consumers buying ordinary goods, green consumers pay more attention to environmental issues [41]. However, environmental concern is not a necessary condition for pro-environmental behavior such as low-carbon consumption [33]. The low-carbon awareness and behavior of colleagues, friends, and relatives significantly influence people [42]. People turn to society for guidance before acting [43]. Schultz et al. (2007) have shown that households with above-average electricity consumption will voluntarily reduce their electricity consumption after receiving information from the community average, while households with below-average electricity consumption will start to use more electricity, like the ’boomerang effect’ [9]. Adverse information has a negative effect on individuals’ low-carbon behavior, causing individuals who previously engaged in low-carbon, energy-efficient, and environmentally friendly behavior to abandon their low-carbon consumption behavior [9]. When people realize the discrepancy between their current behaviors and norms, they will adopt adaptive behaviors [44]. It means that low-carbon consumption behavior is related to the feedback information of other people’s purchase decisions. Information is the most direct tool for promoting consumer purchases of green products, such as social media information [45] and advertising information [46].

Feedback information is defined as an outcome of an action that can be captured by the senses, like vision and audition [47]. In the learning-related field, feedback has been widely investigated since it is crucial for correcting, monitoring, and improving performance [48,49]. Previous studies have suggested that feedback may function as an instruction [50], a rule [51], a motivational [52], or an establishing stimulus [53]. Individuals will encode the information about the group and cumulate it in their memory systems. Then, the prototypical representation, namely a central tendency of the group, which can be treated as descriptive norms, is formed [54]. In this way, the feedback information about the group purchase decision links their low-carbon consumption behavior with descriptive norms. It can be inferred that feedback should be an essential factor in social norms.

The majority of existing research has focused on internal factors that influence low-carbon behaviors, such as individual norms [55]. Some scholars in the field of low-carbon consumption have also confirmed that positive behaviors from consumers’ surroundings will drive their energy-saving behavior [56,57], promote the purchase of environmentally friendly goods, promote low-carbon travel [58], and increase the willingness to pay for carbon emissions [59]. However, there are distinctions between intention and behavior. There are some deficiencies in the relationship between social norms and feedback. The internal mechanisms of feedback that make descriptive norms affect behavior should be studied in detail. Especially, the negative feedback is less discussed.

In this research, we will conduct an in-depth study of the influence of the descriptive norms and the feedback information on individual shopping results through behavioral experiments. It has shown significant correlations between descriptive norms and feedback [60]. Combining the existing findings, we assume that descriptive norms should have an influence on individual purchasing behavior through the feedback information, both positive and negative, of others’ purchase decisions. But the impact of different feedback may be asymmetric.

**H1:**  *Descriptive norms significantly affect one’s low-carbon purchasing behavior by purchasing feedback information, especially negative feedback*.

#### 2.1.2. Injunctive Norms

In addition to descriptive norms, some researchers have found that injunctive norms impact consumers’ buying intentions [61] and promote awareness of the environment. It can motivate consumers to purchase environmentally friendly products [62] and save more energy [63]. Unlike descriptive norms, the messages of injunctive norms are prescriptive and socially approved or disapproved [64]. Injunctive norms are generally understood to mean behaviors that one follows and expects others to follow as well [65].

The interventions of social norms are policy instruments to change people’s behavior [15,66,67]. Conversely, government policy is a type of social norm signal since it reveals social desirability [68]. Policies that guide and restrict people’s behavior could be a kind of enhanced injunctive norm. Accordingly, injunctive norms are usually related to policies [68]. There is an implication of injunctive norms that contravening the code of conduct will be opposed and punished by society and public morals [69]. People tend to recognize and reward ethical behavior or give negations and punishments for substandard actions [70,71]. Previous studies suggest that injunctive norms have less effect on individuals’ behavior than descriptive norms [72,73,74]. It is because these studies about injunctive norms have mostly taken the form of suggestions or prohibitions with no punishment for individuals. As per the focus theory of normative conduct mentioned above, the effect depends on which social norms are more salient. Compared to the reality surrounding pressure, the injunctive norms are weakened by the absence of punishment, so that descriptive norms affect individuals’ behavior to a greater degree. Therefore, we deduce that the focus will be transferred to injunctive norms when effective punishments are applied. When the government proposes a policy on low-carbon consumption, it needs to ensure the effective implementation of the policy by taking some measures. In this case, the injunctive norms may have a more salient effect on promoting low-carbon consumption behavior.

So far, research with actual behavior measures has only focused on positive descriptive norms and injunctive norms without punishment. Nevertheless, there is still a lack of negative descriptive norms and effective injunctive norms. Questionnaires and field experiments are their primary methods, with some limitations on the accuracy of variable control and the measure of behavior changes.

**H2:**  *Injunctive norms have a significant influence on one’s low-carbon purchasing behavior. However, when descriptive norms are conflicted with injunctive norms, individual norm-focus will be on descriptive norms if injunctive norms have no punishment or some measures playing the same role as punishment. Namely, descriptive norms have a more substantial influence on personal low-carbon purchasing behavior*.

### 2.2. Personal Carbon Allowances and Policy Implementation Efficiency

Most previous policy studies emphasize industrial enterprises from a macro-scale perspective, such as motor fuels and electric power [75,76], rather than a micro-scale perspective of individual residents. The policy on enterprises inspired the thoughts of individual low-carbon policies [77]. Some study findings confirmed that the government had a significant influence on individual green behaviors [24]. The development of low-carbon policies for personal consumption seems to have become an irreversible trend. When the personal carbon allowance (PCA) was put forward as a policy proposal to limit individual carbon emissions, a heated debate arose.

The original PCA was defined as a policy proposal that can be traced back to the 1990s [78]. There are multiple forms and names of PCAs, such as the electronic tradable energy quotas in the UK [79] and the tradable transport carbon permits for private transport in France [80]. New, more aspiring PCA suggestions cover economy-wide emissions connected to food, services, and consumption-related carbon emissions [81]. As envisaged, allowances would be withdrawn from personal budgets with each payment for transportation fuel, home heating fuels, and electricity bills [82].

The personal carbon allowances (PCAs) policy provides an economic stimulus and facilitates the development of social norms on low-carbon behavior [83]. PCAs output injunctive information and aim to evoke individual cognitive awareness of carbon, engender carbon consciousness, and impact consumption behavior [83,84]. It seems like social norms and individual low-carbon behavior interact under the framework of PCAs. However, what is not yet clear are the interlinked mechanisms.

According to the above, if the PCA policy can be effectively implemented, it will be conducive to forming social norms on low-carbon consumption and promoting personal low-carbon consumption behavior. Policies play an important role in the impact of norms on individual low-carbon behaviors [85]. Effective policy implementation is affected by many factors, such as policy design, institutional constraints, annual government performance, etc. [86]. Anthoff and Hahn (2010) proved that the efficiency of policy regulation could be obtained by reviewing the data [87]. It means that the effectiveness of policy implementation can be measured. Due to the relationship between policy and injunctive norms and the impact of policy efficiency on emission reduction, this study considers different levels of policy implementation efficiency. We focus on the relationship between injunctive norms and individual low-carbon behavior under high or low policy implementation efficiency.

### 2.3. Public Good Games

Public goods games (PGGs) serve as a valuable means of investigation, both in experimental settings and theoretical models, by allowing participants to equally share a public resource regardless of their individual contributions [88]. The research on public goods shows that the cooperation rate of individuals is closely related to two factors: the cooperation conditions and the punishment on free riders [89,90,91], which correspond to the descriptive norm and injunctive norm, respectively. The condition of cooperation can be the cooperative behavior of others, the reward for collaborators [92], or the punishment for uncooperative people [93]. Therefore, revealing the relationship between reward and punishment may help better understand social norms [94]. Since each low-carbon consumption behavior can benefit the group, every member of the group has free-riding motivation. After establishing the collaborative objectives, whether the cooperation of others meets the expectations will affect the individual’s behavior [95]. The members will have no desire to provide costs for maintaining public goods if they are only concerned with themselves [96]. In this case, the implementation of social norms is based on the premise that violations of the social norms will be punished [97].

Due to these observations, one of the primary goals of this research is to examine the effects of injunctive norms versus descriptive norms on individual behavior. When the injunctive norm can penalize individuals, the reason for their behavioral changes should be analyzed. Hence, the hypothesis is proposed as follows:

**H3:**  *Injunctive norms with high policy implementation efficiency have a more vigorous promotion of low-carbon consumption behavior than descriptive norms. Injunctive norms as a target norm focal can be activated through implementing relative policy effectively. The norm focus will transfer from descriptive norms to injunctive norms*.

## 3. Materials and Methods

The core of our social norms task was a low-carbon purchasing task, underlying a variety of “multistage group decision-making task” paradigms [98]. PCA has been proven to be a meaningful policy instrument in some people’s lifestyles [99]. It was an effective way to cut emissions by establishing relevant policies. Previous studies had demonstrated that carbon footprint calculators could measure personal carbon emissions related to household items like electronic devices or furniture [100]. It meant the policy of PCA could be implemented. According to PCA, each participant will have a carbon quota account. When shopping, in addition to paying the corresponding amount of purchased products, the carbon quota used for by-products will be deducted from their carbon quota account accordingly. Individuals with insufficient carbon quotas can buy others’ excess carbon quotas from the carbon quota market. Based on the personal carbon allowance, we set up a low-carbon consumption context. Whether the policy worked depends on the equity principles and the specific quota numbers. Therefore, to avoid these influences as best as possible, we control the carbon quota strictly. The subjects were informed that their fixed monthly carbon quota for common goods was exhausted in these scenario-based experiments. We also set up four additional virtual consumers whose carbon quota was used up as participants.

The purchasing policy was defined as banning the purchase of ordinary goods when the carbon quota was exhausted. The people without carbon quotas were required to buy extra quotas if they wanted to buy something. Injunctive norms were displayed by this policy of low-carbon consumption in the experiment. Descriptive norms were presented by showing the others’ purchases of low-carbon products. The participants would then make purchasing decisions based on the information on the screen. The effects of social norms were compared by measuring low-carbon goods’ purchase rates in different cases. Participants received a RMB 50 show-up fee and the opportunity to win partial credit for their social practice course. We divided the influence of social norms into the following situations: We researched the influence on the subjects by simulating two kinds of norms through feedback and policy, respectively. To examine the effects of descriptive norms and injunctive norms on low-carbon purchasing behavior, we conducted Experiment 1. Subsequently, to further investigate the effect of policy implementation efficiency on low-carbon purchasing behavior, we designed Experiment 2. Therefore, we conducted two experiments via MATLAB to control the process.

In recent years, higher education institutions have implemented sustainable development initiatives, leading to an increased awareness of environmental issues among university students [101]. Furthermore, university students are known for their active consumer behavior, making them an appropriate demographic for our experimental setup. Therefore, we chose university students as our sample group. Our objective was to gather over 80 valid samples for each experiment. To account for potential last-minute cancellations by registered participants, we allowed more than 100 participants to sign up. Ultimately, 102 and 101 participants, respectively, participated in the experiments. We securely stored the data of all participants and conducted analyses using the valid data collected.

### 3.1. Experiment 1

#### 3.1.1. Participants

A total of 102 healthy university students (including undergraduate and graduate students) were invited to participate in the experiments for monetary reward. All data were collected in May 2021 using MATLAB. Two participants were excluded for their poor performance because they did not seriously consider how to make decisions during the experiment. Additionally, their consistently, exceptionally short response times in each round confirmed this. The final sample consisted of 100 participants (48 females, 19 to 30 years).

All participants in both studies signed consent for confirmation before the experiment. The internal review board of management of Harbin Institute of Technology approved the experiment.

#### 3.1.2. Experiment Design

**Figure 1:**
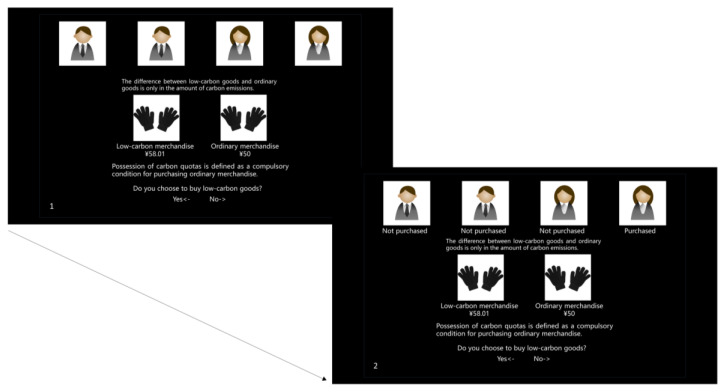
Experiment 1.

Experiment 1 was a 3 (no feedback/positive feedback/negative feedback) × 2 (no policy/policy without the compulsory purchase of carbon quotas) within-subjects designed experiment. It had four blocks. For each experimental block, participants needed to complete 24 trials. The goods were defined as a common type and a low-carbon type, respectively. The participants would be asked to choose the good twice in each trial, based on two different information stimulations. In the experiment, we would randomly show the purchasing choice of the four virtual characters to the subjects in some trials. Here, we have informed the participants that these four purchasers in the picture were virtual characters. If most virtual characters chose low-carbon goods, one descriptive norm was formed through positive feedback. The converse of this arrangement meant that the other descriptive norm would be influenced by subjects’ negative feedback. For participants, portrait photos of the other four virtual characters were always displayed within trials (Figure 1). This manipulation further increased the attraction of the task while creating a more natural situation for the participants. In addition, there was a policy that it was forbidden to keep buying ordinary goods after the carbon quota was exhausted. If the policy existed in the present trial, the relevant information would appear on the screen as a sign to participants. Some trials without feedback and policy were used as controls.

Twenty-four types of university students’ daily consumption merchandise were selected. The prices were all about RMB 50, so the price of ordinary goods was set to be RMB 50, and the low-carbon merchandise price was set as RMB 57.5 with a +/−1.5 fluctuation range. At the beginning of each trial, we gave the subjects a shopping budget of RMB 70. We set the price of low-carbon goods to be 15% higher than that of ordinary goods on average in case the participants who had a RMB 70 budget per trial felt forced to choose ordinary goods due to the price differences. To simulate the actual purchasing decision situation, the surplus from each trial was related to their final income. Participants were told that they needed to spend the RMB 70 budget as if it were their own money and to make decisions based on what they normally consider when spending. In order to prevent the participant’s choices from being influenced by the amounts, the remaining amounts after each trial of choices were not displayed in the experiment. The purpose of this move was simply to immerse the participants in the experimental situation and to prevent them from treating the budget as a windfall and making decisions that were not their true choices. The experiment was not deceptive to the participants. The order of presentation of all conditions is counterbalanced.

#### 3.1.3. Procedure

In the preparation phase, we asked the participants to select six highest and six lowest carbon emissions merchandise from the 24 goods according to their cognition. The last 12 unselected merchandise would be used in the form of common goods and low-carbon goods. We did this to decrease the influence of the nature of the merchandise. We found that the participants considered the carbon emissions of goods in the preliminary experiments.

Before the formal experiment, there were 12 practice trials. The low-carbon purchasing task contained two phases. Participants were asked to make two decisions for one product in a trial. (i) Phase 1. Initial choice (Behavior 1). Upon presenting two choice options using concrete images, participants were asked to make their initial choice by the left and right arrows on the keyboard corresponding to the positions of the goods images. (ii) Phase 2. Choice adjustment (Behavior 2). After making Choice 1, participants needed to confirm their first decision again when they read the information appearing on the screen. Notably, the information contained whether there existed a policy and the four virtual characters’ choices. Namely, the information could change the participant’s previous decision. For instance, a participant chose the low-carbon good as the first choice without any information about the policy or the virtual characters. Then, in the second choice, he found that ¾ of the virtual characters bought the common good without policy, so he might change to purchase the common good like the other people.

### 3.2. Experiment 2

#### 3.2.1. Participants

101 healthy university students (including undergraduate and graduate students) were invited to participate in the experiments for monetary reward. All data were collected in May 2021 using MATLAB. Thirteen participants were excluded due to part of their data loss being caused by a device fault. The final sample consisted of 88 participants (43 females, 19 to 30 years).

All participants in both experiments gave informed written consent before the experiment. The internal review board of management at Harbin Institute of Technology approved the experiment.

#### 3.2.2. Experiment Design

**Figure 2:**
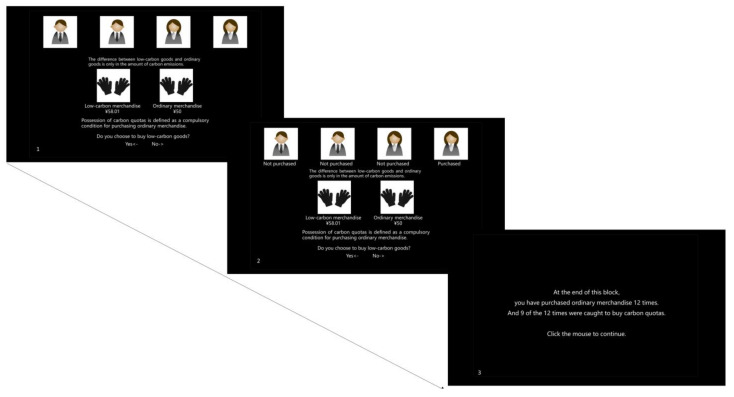
Experiment 2.

In this stage, we conducted a 3 (no feedback/positive feedback/negative feedback) × 2 (low/high policy implementation efficiency). It had two blocks. For each experiment block, participants needed to complete 24 trials. In this part, the policy had two probabilities of efficiency based on the policy of Experiment 1. Experiment 1 did not address the issue of implementation efficiency and only demonstrated the existence of the policy to the subjects. No other measures existed to force subjects to implement the policy. However, in Experiment 2, participants were told that there were supervisors to urge them to ollow the policy. The specific change in low policy implementation efficiency of block 1 was 25%, and the high efficiency of block 2 was 75%. Here, the high policy implementation efficiency means that the rules can be so stringently enforced that people whose carbon quotas are used up have a high probability of being compulsory to buy extra quotas. If the participant insisted on buying ordinary goods, a certain probability would force them to purchase additional carbon quotas. We set a certain high and low probability for the high and low policy implementation efficiency, respectively. The number of times that the participants were caught by supervisors asking to buy carbon quotas will be told to the participants after finishing the corresponding block (Figure 2). Since the participants were only informed of the number of times they were forced to purchase carbon quotas, they were not affected by the amount. And it did not affect the benefits of the participants in the experiment. In experiment 2, the feedback information randomly appeared on the four virtual characters that were the same as in experiment 1. However, unlike experiment 1, all the trials had the PCA policy. The experiment was not deceptive to the participants. The order of presentation of all conditions is counterbalanced.

#### 3.2.3. Procedure

Before the formal experiment, the participants were informed that they could conjecture the efficiency of policy implementation from the practice trial. After the practice, they needed to input the probability level of policy implementation efficiency that they had guessed. For each block, the probability was fixed without experimental deception. The operation of formal experiment 2 was the same as that of experiment 1. At the end of each block, the money used to pay for the extra carbon quotas would be shown to the participants.

## 4. Results

### 4.1. Experiment 1

We recorded participants’ initial and final choices in the experiment. By comparing whether the participants’ choice was changed, we tested whether descriptive norms impact participants’ low-carbon purchasing behavior. If the initial choice and the final choice were different, it indicated that participants were affected by descriptive norms. However, if the initial choice was consistent with the final choice, the descriptive norms could not influence participants’ choices. Therefore, we conducted a Bayesian multinomial logistic regression to analyze whether participants change their behavior under different descriptive norms. We set up four possible outcomes: (1) outcome 1 (baseline): participants stuck to buying an ordinary product in both choices; (2) outcome 2: participants bought a low carbon product in the initial choice and bought an ordinary product in the final choice; (3) outcome 3: participants changed from an ordinary product in the initial choice to a low carbon product in the final choice; (4) outcome 4: participants stuck to choosing the low carbon product in both choices. We set the outcome as the dependent variable, descriptive norms as the independent variable, and participants as the random variable. The result is shown in Table 1.

The coefficient of negative feedback was 2.65, indicating that participants tended to change their initial choice from low-carbon products to ordinary products when they received negative feedback. However, the coefficient of negative feedback was −0.48, suggesting that participants were unlikely to persist in buying low-carbon products while others chose ordinary products. The coefficient of positive feedback was −0.19, which indicated that participants were unlikely to change their initial choice from low-carbon products to ordinary products when others chose low-carbon products. However, the coefficient of positive feedback was 0.54, which proved that participants stuck to choosing low-carbon products when they received positive feedback.

Importantly, we observed that the effect of positive feedback was not significant when participants changed their choice from ordinary products to low-carbon products. The results showed that a negative descriptive norm could prompt participants to give up their prosocial purchasing behavior, while a positive descriptive norm could not make participants give up choosing ordinary products to buy low-carbon products.

To investigate whether injunctive norms impact the participants’ initial choice, we conducted a multilevel logistic regression with injunctive norms as independent variables. The result revealed a significant main effect for injunctive norms (*p* < 0.001 ***). It suggested that injunctive norms could impact the participants’ initial choice significantly.

We also conducted a multilevel logistic regression to ascertain whether injunctive and descriptive norms affected participants’ final choices. We set descriptive norms as the independent variable and participants as the random variable. The result indicated that positive descriptive norms (*p* < 0.001 ***) and negative descriptive norms (*p* < 0.001 ***) affected participants’ choices significantly. Also, descriptive norms significantly affected participants’ low-carbon purchasing behavior (*p* < 0.001 ***). In addition, we calculated the probability of each participant’s purchasing low-carbon products under different conditions. We conducted a mixed linear regression and set the purchasing probability as the dependent variable, injunctive norms and descriptive norms as the independent variable, and participants as the random variable. The result indicated that descriptive norms (*p* < 0.001, η^2^ = 0.35) and injunctive norms (*p* < 0.001, η^2^ = 0.24) significantly affected participants’ purchasing behavior. The effect size indicated that the impact of injunctive norms was weaker than that of descriptive norms.

### 4.2. Experiment 2

We conducted a Bayesian multinomial logistic regression to test whether descriptive norms could change participants’ behavior. We set up four possible outcomes: (1) outcome 1 (baseline): participants stuck to buying an ordinary product in both choices; (2) outcome 2: participants bought a low carbon product in the initial choice and bought an ordinary product in the final choice; (3) outcome 3: participants changed from an ordinary product in the initial choice to a low carbon product in the final choice; (4) outcome 4: participants stuck to choosing the low carbon product in both choices. The result is shown in Table 2.

Under low policy implementation efficiency conditions, the coefficient of negative feedback was 2.24 (*p* < 0.001 ***), indicating that participants tended to change their choice from low-carbon products to ordinary products when they received negative feedback. While the coefficient of positive feedback was 3.85 (*p* < 0.001 ***), suggesting that participants were likely to change their choice from ordinary products to low-carbon products when they found others bought low-carbon products. In addition, the coefficient of negative feedback was −0.48 (*p* < 0.001 ***) and the coefficient of positive feedback was 0.47 (*p* < 0.001 ***), showing that participants were more likely to stick to buying low-carbon products when they received positive feedback and less likely to insist on buying low-carbon products when others sent negative feedback.

Under high policy implementation efficiency conditions, the coefficient of negative feedback was 2.09, proving that participants were more likely to change their initial choice from low-carbon products to ordinary products when they received negative feedback. While the coefficient of positive feedback was 3.22, indicating that participants tended to change their choice from ordinary products to low-carbon products when they found others bought low-carbon products. In addition, the coefficient of negative feedback was −0.25 and the coefficient of positive feedback was 0.47, suggesting that participants tended to persist in buying low-carbon products when they received positive feedback, while they were less likely to do it when they received negative feedback.

Multilevel logistic regression was applied to test whether injunctive norms with efficient implementation could impact participants’ initial low-carbon purchasing behavior. To decompose the effect of high and low policy implementation efficiency, we set four conditions: the first was no injunctive norms (the baseline), the second was injunctive norms without policy implementation efficiency, the third was injunctive norms with low policy implementation efficiency, and the last was injunctive norms with high policy implementation efficiency. We set injunctive norms (i.e., the abovementioned four conditions) as the independent variable, participant purchasing behavior as the dependent variable, and participants as the random variable. The result (shown in Table 3) indicated that low policy implementation efficiency significantly changed participants’ choices (*p* < 0.001 ***), and high policy implementation efficiency significantly affected participants’ choices as well (*p* < 0.001 ***).

To ascertain whether injunctive norms and descriptive norms affected participants’ final choices, a multilevel logistic regression was conducted with injunctive norms and descriptive norms as independent variables and participants as random variables. The result suggested that positive descriptive norms and negative descriptive norms significantly affected participants’ final choices (*p* < 0.001 ***). In addition, the result showed that injunctive norms significantly influenced participants’ final choices (*p* < 0.001 ***).

We conducted a mixed linear regression and set the purchasing probability as the dependent variable, injunctive norms and descriptive norms as the independent variables, and participants as the random variable. The result indicated that descriptive norms (*p* < 0.001, η^2^ = 0.30) and injunctive norms (*p* < 0.001, η^2^ = 0.29) significantly affected participants’ purchasing behavior. The effect size indicated that the impact of injunctive norms and descriptive norms was almost equal.

## 5. Discussion

Social norms are a potent modulator of personal low-carbon behavior [102]. However, how descriptive and injunctive norms interact with behavior in a low-carbon purchasing context awaits further understanding. Here, we bridge this gap with a variety of “multistage group decision-making task” paradigms that allow us to understand the influence mechanisms of social norms. This study indicates that descriptive norms and injunctive norms can effectively improve the purchasing rate of low-carbon goods. Normative focus, the essential part of social norms, has been tested in our experiments. First of all, the experimental data show that both positive and negative feedback can change people’s consumption behavior. Compared with the purchase rate decrease caused by negative feedback, positive feedback can bring a higher increment. Secondly, when there is a conflict between descriptive norms and injunctive norms, the focus of social norms will be concentrated on descriptive norms. At this point, descriptive norms have a more substantial effect on purchasing behavior than injunctive norms, which are just policies without high policy implementation efficiency. Thirdly, the normative focus shifts when the injunctive norm is carried out with high policy implementation efficiency. The increasing policy implementation efficiency encourages the change in the rate of low-carbon purchases brought about by the injunctive norm. In this paper, we identify the pivotal factors, feedback, and policy implementation efficiency needed to change the normative focus and behavior in the context of personal low-carbon consumption. Some researchers have used questionnaires or field experiments to confirm that descriptive norms are an essential factor in changing individual low-carbon consumption behavior [6,8]. Unlike the original methods, we adopted behavioral experiments to prove our hypotheses. Methodologically, this research makes up for the vacancy of behavioral experiments in this field so that we can manipulate and observe the influence of social norms on individual purchasing behavior intuitively.

When people are under conditions of high uncertainty, they conform to descriptive norms [103]. Individuals think that the actions of others may convey relevant information, so it is helpful to imitate the behavior of others, namely following the descriptive norms [104]. In Experiment 1, we found that positive feedback can effectively increase low-carbon product sales volume. Meanwhile, the negative feedback has a counteractive effect. Individual behavior changes with the feedback of others’ behavior, mainly due to the following reasons: Firstly, people tend to associate the feedback of others’ behavior with their own and adjust their behavior according to the feedback to gain recognition [105]. Some classical studies explain the above phenomenon with the relevant theories of conformity [106,107,108]. When an individual holds a different opinion on a specific behavior against the group, social pressure caused by such a difference will influence the individual [109]. Due to the group boundaries function, he/she was inclined to adjust his/her behavior to adapt to the group and effectively reduce the group’s exclusion [110]. Secondly, the above-mentioned typical behavior of the group formed social norms [111]. Social norms are those social phenomena spread among group members through communication. Since it is an essential way of communicating through the feedback information of group members, feedback will have a significant impact on descriptive norms formation [112]. As a kind of social norm, descriptive norms send people a message that makes individuals doubt whether their distinctive behavior is correct [113]. The message is that “the things that the masses do are good ideas”. Self-doubt leads the individual to change behaviors. Descriptive norms provide a standard for feedback information that people are reluctant to stray from. Therefore, individual choices in circumstances with no purchasing choice information will change when they get the feedback of others. Namely, descriptive norms change individual behavior through feedback information. Thirdly, green consumption can bring long-term advantages to humans, but it also necessitates everyone’s participation [114]. In other words, the environmental influence of each individual’s activity is negligible. However, their prosocial behavior will be significantly impacted when numerous people do the same things [115]. Therefore, when people need to choose between low-carbon goods and common goods, they will consider others’ purchasing decisions. It can help them judge whether their choice of low-carbon goods effectively improves the environment or not. When a consumer’s choice is different from the descriptive norms formed by the dominant feedback, he will face alienation, referring to a negative feeling of being separated from others [116]. In the context of low-carbon consumption, alienation can bring powerlessness and meaninglessness that make consumers think their low-carbon behavior has little influence and cannot contribute to the goal of environmental improvement. The conflict between behavior and feedback, especially negative feedback, indicates that not everyone is taking action, making people feel ineffective and futile [117]. Alienation can even make consumers unable to recognize the environmental value and importance of their green consumption practices [118]. It indicates the importance of everyone participating in low-carbon consumption.

Another phenomenon in Experiment 1 is that the improved low-carbon goods purchase rate caused by positive feedback is more significant than the reduction brought by negative feedback. It means that different feedback information has different effects on personal purchasing decisions. The two contradictory feedbacks make people face information conflict, creating confusion about what is authentic and appropriate [119]. The subjects knew the importance of low-carbon consumption according to the experimental context. They understood that buying low-carbon goods is a behavior needed by the current society. Positive feedback about others’ purchasing behavior that is consistent with social needs will be more helpful in activating personal social norms on low-carbon consumption because social norms are the source of individual behavior motivation [115]. In environmental protection, some studies on waste recycling and water conservation fully support the above explanation [120,121]. Individuals are stressed over the positive feedback information that makes them act according to the norms. Therefore, the feedback information consistent with social norms is more effective than the opposite feedback [122,123]. In our experiment, negative feedback contradicts the social demand for people’s low-carbon behavior. Feedback produces the motivation that makes individuals eliminate the difference between their behaviors and the feedback information. At this point, the feedback information intensity affects consumers’ purchase intentions. In learning feedback information, there are different levels of behavioral change [47]. Thus, the effects of two kinds of opposite feedback information on individual behavior are asymmetric.

In terms of injunctive norms, when there is only feedback or policy with no effective implementation, feedback can improve the low-carbon consumption rate to a greater extent than policy. Because, compared with injunctive norms, descriptive norms can enhance the preference for prosocial behaviors, including low-carbon behaviors [124]. In the absence of effective policy implementation, the effect of injunctive norms is weaker than that of descriptive norms on increasing the rate. In addition, when descriptive norms and injunctive norms conflict, the impact of descriptive norms is stronger than that of injunctive norms as well. In making a decision, whether for the approbation of social norms or gaining approval from group members, people will adjust their behavior to match the social norms by learning from others’ behavior [125]. According to the focus theory of normative conduct, neither descriptive nor imperative norms are likely to affect behavior unless it becomes the normative focus [126]. The impact of the two kinds of norms on individual behavior is determined by whether the norms are prominent in individual cognition [119]. As the descriptive norm is a significant factor in low-carbon consumption, it has a substantial effect on individual behavior. Thirdly, for individuals who have chosen low-carbon goods, descriptive norms can effectively make them change to ordinary goods through negative feedback. The boomerang effect of negative feedback undermines the descriptive norm established by positive feedback [9]. However, Schultz et al. (2007) also noted that adding injunctive norms can effectively prevent the boomerang effect. Our experiments confirmed their views as well. When negative feedback and policy exist simultaneously, the extent of the low-carbon commodity purchase rate reduced by negative feedback is less than that without policy. Last, we observed that descriptive and injunctive norms had a better effect on promoting low-carbon consumption when these two kinds of norms were coincident. The purchase rate of low-carbon goods became higher in this case. It also supports the conclusion that presenting consistent norms can lead to greater behavior changes than only one type of norm [127].

Experiment 2 enriched the content of injunctive norms. When the other conditions are fixed, the high efficiency of policy implementation can significantly improve the low-carbon consumption rate. We analyze the following reasons for this phenomenon: First and foremost, the high policy implementation efficiency leads to a shift in normative focus. In the above analysis of the conflict between descriptive norms and injunctive norms, we confirmed that descriptive norms had stronger effects. The specific feedback provides a template for norms to compare future behaviors. However, the behavioral changes may be temporary, and the formation of descriptive norms depends on the context [120]. When a policy has a high level of potential to be implemented effectively, individual injunctive norms are activated by implementation efficiency. Golman et al. (2017) pointed out that receiving information on injunctive norms may lead individuals to avoid future “judgmental” information to respond to the review or social judgment [128]. It means that people ignore the potential for effective feedback that can be used to adjust their behaviors because of information avoidance. At this point, individuals pay more attention to injunctive norms, which become the normative focus of policy implementation. Therefore, despite the conflict between descriptive norms and injunctive norms, the people who initially followed the descriptive norms to buy ordinary goods changed to obey the injunctive norms in consideration of policy. Moreover, the behavioral changes generated from activating injunctive norms are broader and longer-lasting than those of descriptive norms [69].

Secondly, the externality of low-carbon behaviors makes individuals understand that all the group members will enjoy the benefit even though only some carry out the low-carbon consumption. In “prosocial” decision-making, people may shift their attention from themselves to others. Individuals will respond positively to those who contribute to the collective interest [129]. Whether others are involved in low-carbon consumption or not becomes a condition for individuals to make their decisions. This reflects the degree of cooperation among group members. The PCA policy in the experiment requires individuals whose carbon emissions exceed the standard to purchase additional carbon quotas. It indicates that people need to pay more for ordinary goods because of effective policy implementation. The high policy implementation efficiency becomes a disguised punishment for individuals whose carbon emissions exceed the standard. Punishment can promote more stable cooperation among group members [130]. Therefore, when the level of policy implementation efficiency increases, individuals tend to think that the degree of cooperation increases because more people will buy low-carbon goods to avoid such punishment. The individual purchase rate of low-carbon goods increases as well.

Thirdly, for the same reason as the externality, each person in the context has the possibility of a free ride. While promoting low-carbon behaviors, third-party punishment is necessary for social norms to ensure fairness. As a public resource, the environment that low-carbon behaviors can improve is beneficial to everyone. People who do not choose low-carbon goods also enjoy the rewards brought by environmental change. It will make the people who purchase low-carbon goods perceive unfairness. It has been proposed that the preference for fairness is a sensitive factor in decision-making [131,132]. The behavior mechanisms of social norms and rewards are similar to those of social equity decision-making [133]. The psychological change from fairness plays a vital role because the aversion to unfairness can impact the implementation effect of social norms [134,135]. As the policy reveals social desirability, the policy is a social norm signal [68]. Under PCA circumstances, people with excessive carbon emissions violate social norms and have to use money to buy extra carbon quotas, making other individuals feel fair. To maintain this kind of fairness and ensure the balance of the social group, high policy implementation efficiency is required, which means an injunctive norm with punishment.

## 6. Conclusions

In this study, feedback and policy, as crucial factors affecting social norms, are the keys to changing personal low-carbon purchasing behavior. Firstly, we have confirmed H1, which states that descriptive norms influence individual low-carbon consumption behavior through feedback information. Even though the participants told us that they did not care about the outcome of others’ purchases, they did unconsciously change their purchase decisions. Secondly, we have also validated H2: that injunctive norms do have a facilitative effect on the purchase of low-carbon goods, but this effect is strongly reduced when the descriptive norms are reversed. Furthermore, we confirmed H3, indicating that high policy implementation efficiency is more likely to make people respond to injunctive norms to change their behavior. When individuals can clearly experience the punishment associated with injunctive norms, injunctive norms have a greater promoting effect on individual low-carbon purchasing behavior. The application of social norms can effectively increase low-carbon consumption behavior.

The present study has important theoretical significance. It not only provides empirical evidence and recommendations for the focus theory of normative conduct, but it also introduces new variables, such as feedback information and punishment, and expands the framework and application of the framework. In addition, unlike previous survey methods, such as questionnaires and field experiments, we use behavioral experiments, which considerably raise the reliability and validity of the results. The representativeness and objectivity of the data are more accurate. In terms of policy, we offer insights and recommendations to policymakers. The findings suggest an important policy tool to achieve low-carbon development. When public policymakers are formulating the plans, they should strengthen the feedback on people’s low-carbon consumption status and improve related policies’ implementation efficiency as well. We provide evidence for policymakers to better utilize social norms in guiding individual behavior. Particular attention should be paid to the effectiveness of policy implementation. More stimulating and normative policies should be implemented to increase the flexibility of the regulatory system and encourage low-carbon behavior through stimulation and regulation. The government can also organize lectures and other promotional activities on low-carbon consumption for residents, using social norm information to raise individuals’ environmental awareness and change people’s consumption habits. In terms of business, we provide guidance and suggestions for companies. Companies can utilize relevant social norm information based on government policies or the purchasing behavior of others to promote their products and attract consumers to make purchases. 

Although this study makes up for some gaps in this field, there are still some limitations. The social distance between the feedback information providers and the individuals may have an influence on their purchase behaviors. In addition, the amount of punishment or penalties related to personal financial interests can also influence individual behavior decisions. Therefore, we will continue to study these specific contents, combining psychology, sociology, and economics. In practical applications, there are still challenges in implementing PCA policies. Further research is needed to establish specific system frameworks for PCAs and implementation guidelines.

## Figures and Tables

**Table 1:** The outcome of multinominal Bayesian logistic regression (Experiment 1).

	Estimate	Est. Error	l-95% CI	u-95% CI	p (MAP)
low carbon product to ordinary product					
Intercept	−4.52	0.25	−5.02	−4.04	<0.001 ***
negative feedback	2.65	0.16	2.34	2.98	<0.001 ***
positive feedback	−0.19	0.28	−0.76	0.34	0.015 *
ordinary product to low carbon product					
Intercept	−4.33	0.21	−4.77	−3.93	<0.001 ***
negative feedback	−0.25	0.23	−0.7	0.2	0.75
positive feedback	3.77	0.16	3.45	4.1	0.605
no change in selecting a low-carbon product					
Intercept	−0.44	0.15	−0.73	−0.13	<0.001 ***
negative feedback	−0.48	0.06	−0.6	−0.36	<0.001 ***
positive feedback	0.54	0.06	0.42	0.66	<0.001 ***

Note: * *p* < 0.05; *** *p* < 0.001.

**Table 2:** The outcome of multinominal Bayesian logistic regression (Experiment 2).

	Estimate	Est. Error	l-95% CI	u-95% CI	p(MAP)
low policy implementation efficiency conditions					
low carbon product to ordinary product					
Intercept	−4.06	0.35	−4.79	−3.4	<0.001 ***
negative feedback	2.24	0.22	1.83	2.68	<0.001 ***
positive feedback	0.6	0.28	0.05	1.16	0.107
ordinary product to low carbon product					
Intercept	−4.65	0.38	−5.43	−3.94	<0.001 ***
negative feedback	0.54	0.3	−0.03	1.14	0.208
positive feedback	3.85	0.25	3.4	4.38	<0.001 ***
no change in selecting a low-carbon product					
Intercept	−0.26	0.23	−0.73	0.19	0.534
negative feedback	−0.48	0.09	−0.66	−0.31	<0.001 ***
positive feedback	0.47	0.09	0.29	0.65	<0.001 ***
high policy implementation efficiency conditions					
low carbon product to ordinary product					
Intercept	−3.7	0.29	−4.29	−3.15	<0.001 ***
negative feedback	2.09	0.23	1.65	2.55	<0.001 ***
positive feedback	−0.78	0.48	−1.81	0.07	0.253
ordinary product to low carbon product					
Intercept	−3.49	0.25	−4.01	−3.01	<0.001 ***
negative feedback	−0.18	0.28	−0.74	0.37	0.88
positive feedback	3.22	0.21	2.82	3.64	<0.001 ***
no change in selecting a low-carbon product					
Intercept	0.02	0.14	−0.26	0.31	0.975
negative feedback	−0.25	0.09	−0.42	−0.07	0.022 *
positive feedback	0.52	0.09	0.34	0.7	<0.001 ***

Note: * *p* < 0.05; *** *p* < 0.001.

**Table 3:** The result of multilevel logistic regression (Experiment 2).

	Estimate	Std. Error	z value	Pr (>|z|)
Intercept	−1.192	0.130	-9.153	<0.001 ***
no implementation efficiency	1.671	0.044	38.258	<0.001 ***
low implementation efficiency	1.848	0.062	29.715	<0.001 ***
high implementation efficiency	2.594	0.070	37.159	<0.001 ***

Note: *** *p* < 0.001.

## Data Availability

The data that support the findings of this study are available from the corresponding author upon reasonable request.
